# Systems Biology behind Immunoprotection of Both Sheep and Goats after Sungri/96 PPRV Vaccination

**DOI:** 10.1128/mSystems.00820-20

**Published:** 2021-03-30

**Authors:** Sajad Ahmad Wani, Manas Ranjan Praharaj, Amit R. Sahu, Raja Ishaq Nabi Khan, Shikha Saxena, Kaushal Kishor Rajak, Dhanavelu Muthuchelvan, Aditya Sahoo, Bina Mishra, Raj Kumar Singh, Bishnu Prasad Mishra, Ravi Kumar Gandham

**Affiliations:** a ICAR-Indian Veterinary Research Institute (IVRI), Bareilly, India; b The Ohio State University, Columbus, Ohio, USA; c DBT-National Institute of Animal Biotechnology, Hyderabad, India; d ICAR-Indian Veterinary Research Institute (IVRI), Mukteswar, India; e ICAR-Directorate of Foot and Mouth Disease, Mukteswar, India; Tufts University; School of Animal Biotechnology, Post Graduate Institute of Veterinary Education and Research, Guru Angad Dev Veterinary and Animal Sciences University, Ludhiana, Punjab

**Keywords:** ISG15, PBMCs, Peste des petits ruminants, Sungri/96 PPRV

## Abstract

Immune response is a highly coordinated cascade involving all the subsets of peripheral blood mononuclear cells (PBMCs). In this study, RNA sequencing (RNA-Seq) analysis of PBMC subsets was done to delineate the systems biology behind immune protection of the vaccine in sheep and goats. The PBMC subsets studied were CD4^+^, CD8^+^, CD14^+^, CD21^+^, and CD335^+^ cells from day 0 and day 5 of sheep and goats vaccinated with Sungri/96 peste des petits ruminants virus. Assessment of the immune response processes enriched by the differentially expressed genes (DEGs) in all the subsets suggested a strong dysregulation toward the development of early inflammatory microenvironment, which is very much required for differentiation of monocytes to macrophages, and activation as well as the migration of dendritic cells into the draining lymph nodes. The protein-protein interaction networks among the antiviral molecules (IFIT3, ISG15, MX1, MX2, RSAD2, ISG20, IFIT5, and IFIT1) and common DEGs across PBMC subsets in both species identified ISG15 to be a ubiquitous hub that helps in orchestrating antiviral host response against peste des petits ruminants virus (PPRV). IRF7 was found to be the key master regulator activated in most of the subsets in sheep and goats. Most of the pathways were found to be inactivated in B lymphocytes of both the species, indicating that 5 days postvaccination (dpv) is too early a time point for the B lymphocytes to react. The cell-mediated immune response and humoral immune response pathways were found more enriched in goats than in sheep. Although animals from both species survived the challenge, a contrast in pathway activation was observed in CD335^+^ cells.

**IMPORTANCE** Peste des petits ruminants (PPR) by PPR virus (PPRV) is an World Organisation for Animal Health (OIE)-listed acute, contagious transboundary viral disease of small ruminants. The attenuated Sungri/96 PPRV vaccine used all over India against this PPR provides long-lasting robust innate and adaptive immune response. The early antiviral response was found mediated through type I interferon-independent interferon-stimulated gene (ISG) expression. However, systems biology behind this immune response is unknown. In this study, *in vivo* transcriptome profiling of PBMC subsets (CD4^+^, CD8^+^, CD14^+^, CD21^+^, and CD335^+^) in vaccinated goats and sheep (at 5 days postvaccination) was done to understand this systems biology. Though there are a few differences in the systems biology across cells (specially the NK cells) between sheep and goats, the coordinated response that is inclusive of all the cell subsets was found to be toward the induction of a strong innate immune response, which is needed for an appropriate adaptive immune response.

## INTRODUCTION

Peste des petits ruminants (PPR) is an World Organisation for Animal Health (OIE)-listed acute, highly contagious transboundary viral disease of small ruminants, caused by PPR virus (PPRV) of genus *Morbillivirus* and family *Paramyxoviridae* (1). Morbidity and mortality can be as high as 100% and 90%, respectively (2). The disease manifests as fever, discharge from the eyes and nose, stomatitis, pneumonia, and enteritis (3). PPRV vaccine developed by continuous passage (*n* = 59) of Sungri/96 strain in Vero cells is widely used throughout India (4). The vaccine provides long-lasting robust innate and adaptive humoral immunity and a strong cell-mediated immunity (2), which, however, warrants further investigation (5–7). PPRV is lymphotropic and epitheliotropic (8–10). The primary receptors for PPRV include the signaling lymphocyte activation molecule (SLAM) on activated T cells, B cells, and dendritic cells and the Nectin-4 receptor on epithelial cells (8, 9, 11).

Immune response is complex within a host, and different cell types respond differently to infection, as different classes of receptors receive cues and produce distinct effector molecules (12). It is a highly coordinated effort of distinctly programmed hematopoietic cell types and a product of various direct and indirect effects and interactions between similar or different cell types (13). Moreover, the tissue microenvironment also affects the elicited immune response. In the case of viruses, the complexity of the host response depends on variations in genetic makeup, cell tropism, and replication kinetics (12–15). Peripheral blood mononuclear cells (PBMCs) include T helper cells (CD4^+^), T cytotoxic cells (CD8^+^), B lymphocytes (CD21^+^), monocytes (CD21^+^), natural killer cells (CD335^+^), and dendritic cells (CD320^+^), which play an important role in virus recognition and induce immune response for host defense. While analyzing whole blood or PBMCs, the response of underrepresented cell populations can be masked (13).

Despite advances in our understanding of vaccines, the mechanisms by which protective immune responses are orchestrated among the cell subsets are little known. Molecular patterns and gene signatures detected in blood postvaccination represent a strategy to prospectively determine vaccine efficacy (16). The conventional immunological methods like enzyme-linked immunosorbent assay (ELISA), enzyme-linked immunosorbent spot assay (ELISpot), etc., are of utmost importance in this regard and may continue to remain so in the future (17). However, these approaches are inept at predicting the systems biology behind immune protection. Delineating the systems biology would help in understanding the molecular mechanisms of vaccine-induced immune responses. RNA sequencing (RNA-Seq) is a widely used quantitative transcriptome profiling system for deciphering the systems biology comprehensively (18). Previously, RNA sequencing was used to unravel transcription factors, which modulate the immune response to PPRV Sungri/96 live attenuated vaccine strain *in vitro* in PBMCs (6). Also, a predicted immune signaling pathway of PPRV Sungri/96 vaccine-induced immune response with the predominant role of interferon regulatory factors (IRFs), tripartite motif family (TRIMs), and interferon-stimulated genes (ISGs) in creation of a robust antiviral state *in vitro* in PBMCs has been proposed (7).

Until now there have been no *in vivo* reports of transcriptome profiling of PBMC subsets in PPRV-vaccinated goats and sheep. Herein, transcriptional profiling of circulating CD4^+^, CD8^+^, CD14^+^, CD21^+^, and CD335^+^ cells of PPRV-vaccinated sheep and goats at 0 day (control, i.e., just before vaccination) and before the development of antibody response (5 days postvaccination, i.e., 5 dpv) to decipher the vaccine-induced immune response was done.

## RESULTS

**FIG 1:**
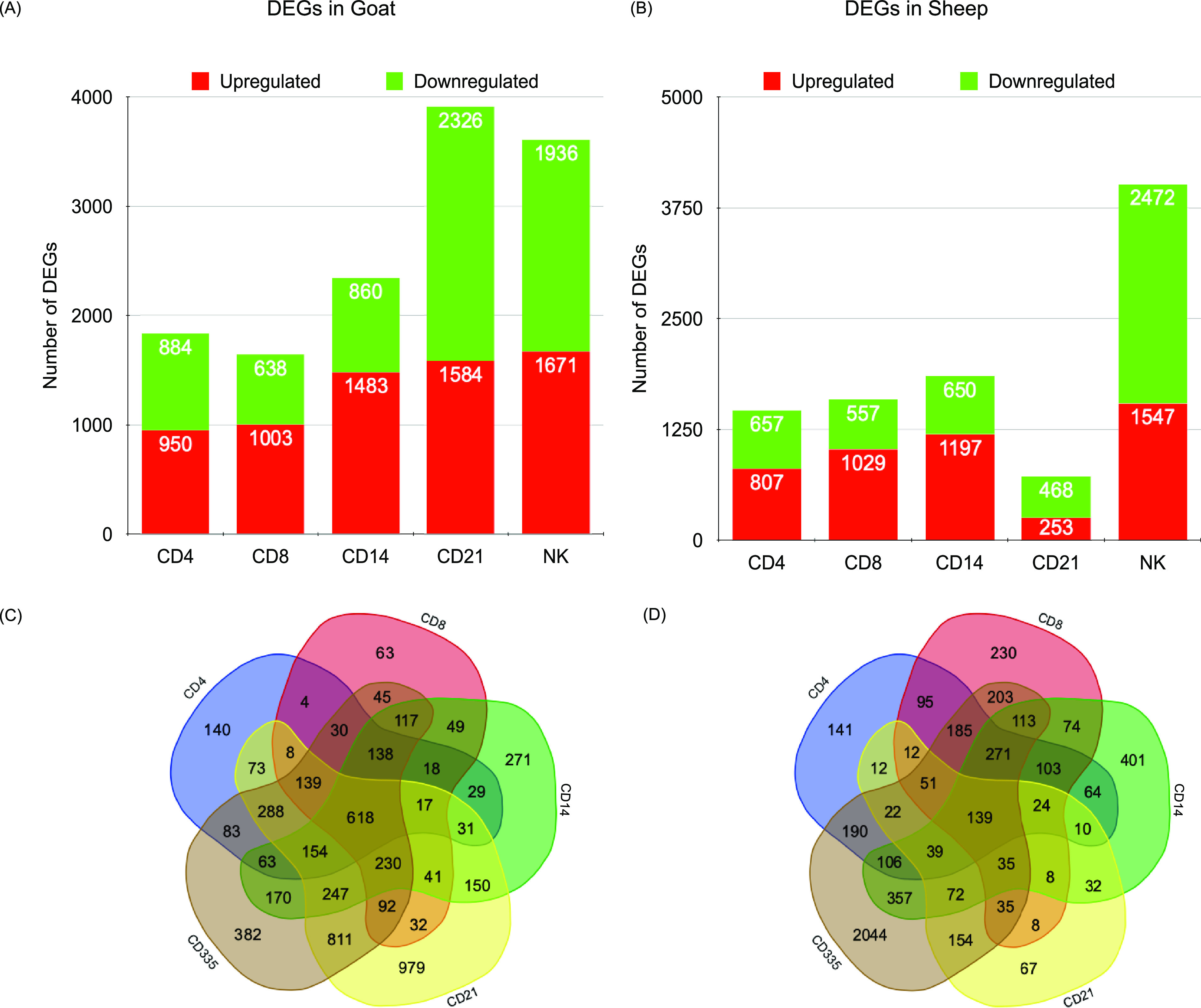
Number of dysregulated DEGs in PBMC subsets in goats (A) and sheep (B). The green color represents downregulation, and the red color represents upregulation. (C and D) Venn diagrams representing unique/common DEGs among cells in goats (C) and sheep (D).

In the present study, CD4^+^, CD8^+^, CD14^+^, CD21^+^, and CD335^+^ cells were enriched (see Fig. S1 in the supplemental material) from the blood collected (five goats and five sheep) at 0 day and 5 dpv (5 days postvaccination). RNA was isolated from these subsets to profile the transcriptome to delineate the systems biology behind the Sungri/96 vaccine-induced immunoprotection at 5 dpv in sheep and goats. The number of differentially expressed genes (DEGs) in CD4^+^, CD8^+^, CD14^+^, CD21^+^, and CD335^+^ cells were 1,834, 1,641, 2,343, 3,910, and 3,607, respectively, in goats and 1,464, 1,586, 1,847, 721, and 4,019, respectively, in sheep (Fig. 1A and B). Venn diagram was generated to examine the common and unique DEGs among cells. In comparison, 618 and 139 DEGs were found in common among CD4^+^, CD8^+^, CD14^+^, CD21^+^, and CD335^+^ cells, in goats and sheep, respectively. The number of unique DEGs was highest in the CD21^+^ cells of goats and CD335^+^ cells of sheep (Fig. 1C and D).

10.1128/mSystems.00820-20.1 FIG S1 Purity of PBMC subsets, T helper cells (CD4^+^), T cytotoxic cells (CD8^+^), monocytes (CD14^+^), B lymphocytes (CD21^+^), and natural killer cells (CD335^+^) by flow cytometry. **“**PBMCs” means unstained cells. “PBMCs with antibody” means before magnetic bead cell separation. “Enriched cells” means after magnetic bead cell separation. Download FIG S1, PDF file, 1.0 MB. Copyright © 2021 Wani et al. 2021 Wani et al. https://creativecommons.org/licenses/by/4.0/ This content is distributed under the terms of the Creative Commons Attribution 4.0 International license.

### Gene Ontology analysis.

**FIG 2:**
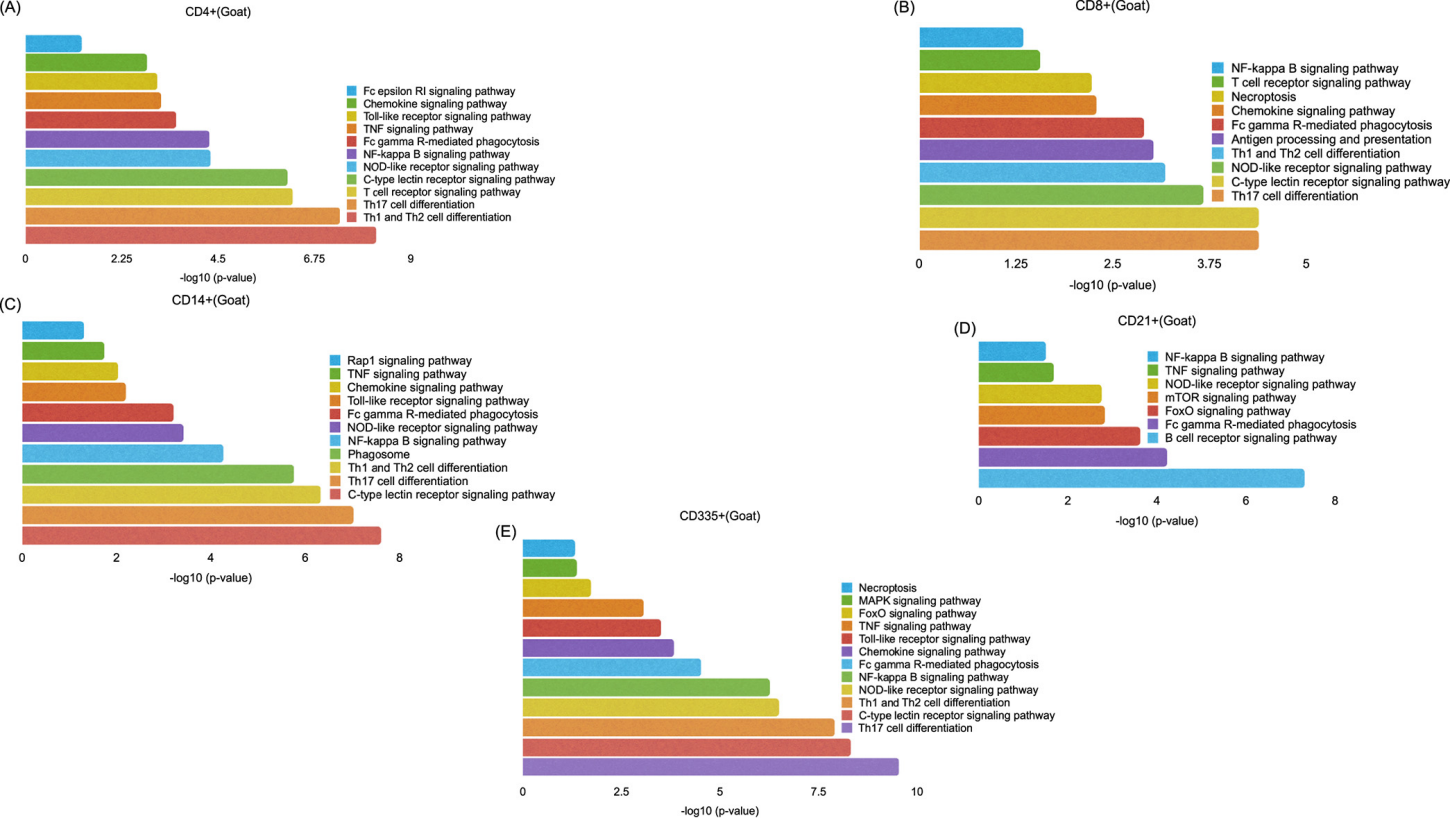
Functional annotation of DEGs involved in immunological processes for each subset of PBMCs, CD4^+^ (A), CD8^+^ (B), CD14^+^ (C), CD21^+^ (D), and CD335^+^ (E) cells using g:Profiler in goats.

**FIG 3:**
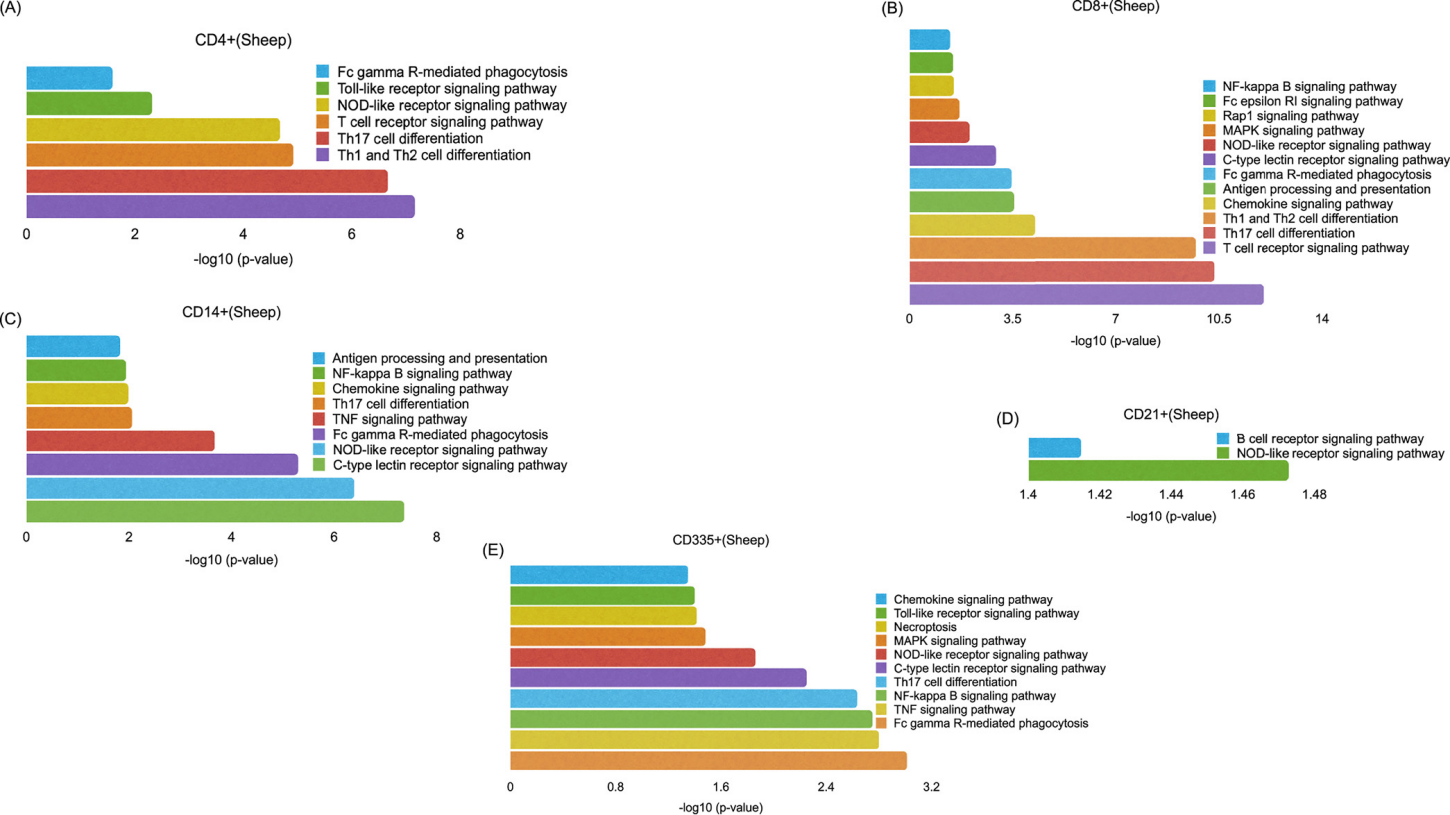
Functional annotation of DEGs involved in immunological processes for each subset of PBMCs, CD4^+^ (A), CD8^+^ (B), CD14^+^ (C), CD21^+^ (D), and CD335^+^ (E) cells using g:Profiler in sheep.

Initially to evaluate the changes within a subset, the functional annotation for genes expressed in each subset was done using g:profiler. The immune system Kyoto Encyclopedia of Genes and Genomes (KEGG) pathways enriched in each subset were assessed. In all the cells, an innate immune response leading to cell-mediated adaptive immune response was observed (Fig. 2 and 3). For comparison across subsets between species, please refer to Fig. S2.

10.1128/mSystems.00820-20.2 FIG S2 Heatmap depicting the significance values (−log_10_ *P* value) of pathways enriched among the subsets in both the species. The red color goes from the least value to the highest value in lighter to darker shade. Download FIG S2, PDF file, 0.03 MB. Copyright © 2021 Wani et al. 2021 Wani et al. https://creativecommons.org/licenses/by/4.0/ This content is distributed under the terms of the Creative Commons Attribution 4.0 International license.

### (i) CD4^+^ cells of sheep and goats.

On comparing CD4^+^ cells in sheep and goats, Fc gamma receptor (Fc gamma R)-mediated phagocytosis, Toll-like receptor signaling pathway, NOD-like receptor signaling pathway, Th1 and Th2 cell differentiation, T-cell receptor signaling pathway, and Th17 cell differentiation were found significantly enriched in both species. Besides these, in goats, Fc epsilon RI signaling pathway, C-type lectin receptor signaling pathway, tumor necrosis factor (TNF) signaling pathway, chemokine signaling pathway, and NF-κB signaling pathway were found enriched in CD4^+^ cells.

### (ii) CD8^+^ cells of sheep and goats.

In CD8^+^ cells, Th17 cell differentiation, C-type lectin receptor signaling pathway, NOD-like receptor signaling pathway, antigen processing and presentation, Th1 and Th2 cell differentiation, T-cell receptor signaling pathway, Fc gamma R-mediated phagocytosis, chemokine signaling pathway, NF-κB signaling pathway were found enriched in both sheep and goats. Besides these, three more pathways: Rap1 signaling pathway, Fc epsilon RI signaling pathway, and mitogen-activated protein kinase (MAPK) signaling pathway were enriched in sheep.

### (iii) CD14^+^ cells of sheep and goats.

In CD14^+^ cells of both species, Th17 cell differentiation, C-type lectin receptor signaling pathway, NOD-like receptor signaling pathway, TNF signaling pathway, Fc gamma R-mediated phagocytosis, chemokine signaling pathway, and NF-κB signaling pathway were found enriched. Additionally, in goats, the Toll-like receptor signaling pathway, Rap1 signaling pathway, and phagosome Th1 and Th2 cell differentiation were enriched.

### (iv) CD335^+^ cells of sheep and goats.

In CD335^+^ cells, Th17 cell differentiation, Toll-like receptor signaling pathway, C-type lectin receptor signaling pathway, necroptosis, MAPK signaling pathway, NOD-like receptor signaling pathway, TNF signaling pathway, Fc gamma R-mediated phagocytosis, chemokine signaling pathway, and NF-κB signaling pathway were enriched in sheep and goats (Fig. 2 and 3). Th1 and Th2 cell differentiation and FoxO signaling pathway were found enriched additionally in goat CD335^+^ cells.

### (v) Common DEGs in each subset between sheep and goats.

The common genes in sheep and goats that are involved in immunological processes in each subset are represented in a heatmap (Fig. S3). In CD4^+^, CD8^+^, CD14^+^, CD21^+^, and CD335^+^ cells, the numbers of common genes involved in immunological processes were found to be 67, 91, 122, 17, and 179, respectively. Most of the common DEGs in CD4^+^, CD8^+^, and CD14^+^ cells were found upregulated in both species. However, in CD21^+^ and CD335^+^ cells, a contrast in the expression of these genes was observed between sheep and goats. Most of the DEGs were upregulated in goats but downregulated in sheep.

10.1128/mSystems.00820-20.3 FIG S3 Heatmap for fold change (Log_2_FC) value of common immune DEGs between goats and sheep for each subset of PBMCs, (A) CD4^+^, (B) CD8^+^, (C) CD14^+^, (D) CD21^+^, and (E) CD335^+^. The green color indicates downregulation, and the red color indicates upregulation. Download FIG S3, PDF file, 0.3 MB. Copyright © 2021 Wani et al. 2021 Wani et al. https://creativecommons.org/licenses/by/4.0/ This content is distributed under the terms of the Creative Commons Attribution 4.0 International license.

### (vi) Coordinated response.

To understand the coordinated response across all the subsets, genes expressed in all cell subsets were functionally annotated. A total of 5,512 and 5,297 genes were found expressed across all subsets (see Data Set S2 in the supplemental material) in goats and sheep, respectively. Among these, in goats and sheep, 689 and 703 genes, respectively, were found associated with innate immune response biological processes (Fig. S4). A subset of 544 immune response genes was found to be common between sheep and goats with 144 and 158 genes being unique, respectively. This shows that in both sheep and goats, the coordinated vaccine response at 5 dpv across all the subsets is toward triggering a strong innate immune response, as evident from the upregulation of innate immune genes.

10.1128/mSystems.00820-20.4 FIG S4 Functional annotation related to immunological processes of genes expressed in all the subsets or in one subset of PBMCs associated with innate immune response in (A) goats and (B) sheep. (C) Heatmap depicting the significance values (−log_10_ *P* value) of biological processes enriched in both the species. The red color goes from the least value to the highest value in lighter to darker shade. Download FIG S4, EPS file, 1.2 MB. Copyright © 2021 Wani et al. 2021 Wani et al. https://creativecommons.org/licenses/by/4.0/ This content is distributed under the terms of the Creative Commons Attribution 4.0 International license.

10.1128/mSystems.00820-20.6 DATA SET S2 List of 5,512 and 5,297 DEGs expressed across the subsets in goats and sheep, respectively. Download Data Set S2, XLS file, 1.2 MB. Copyright © 2021 Wani et al. 2021 Wani et al. https://creativecommons.org/licenses/by/4.0/ This content is distributed under the terms of the Creative Commons Attribution 4.0 International license.

### Comparison analysis across subsets using Ingenuity Pathway Analysis.

**FIG 4:**
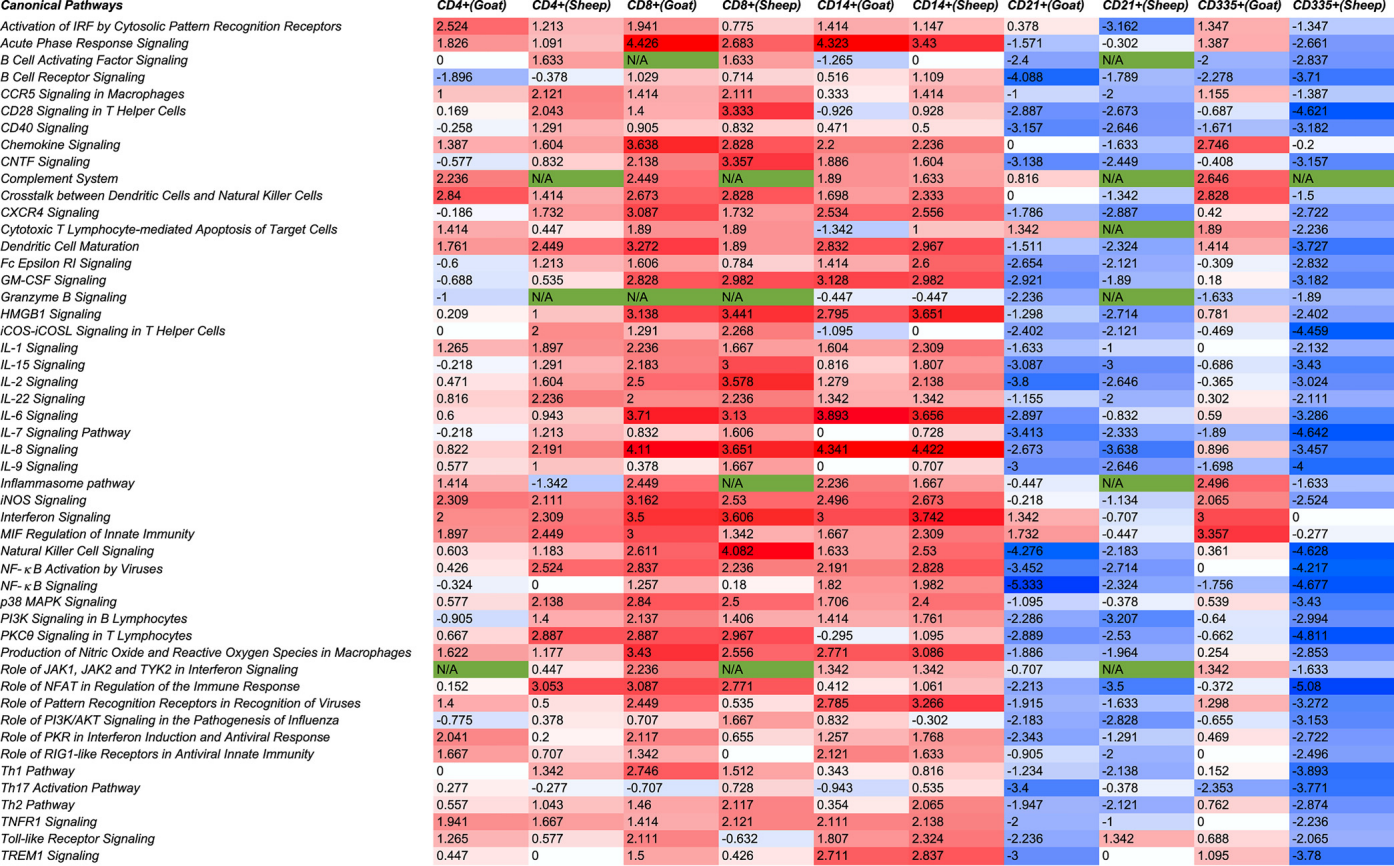
Comparison analysis of canonical pathways related to immunological processes among the subsets of PBMCs in both sheep and goats using IPA. Blue color represents a Z score of <0, and red color represents a Z score of >0. A Z score of ≥2 means activation of canonical pathways, and a Z score of less than or equal to −2 means inactivation of canonical pathways. Green color is given to all values not available. The lighter to dark shades indicate increase in mod Z score values.

Ingenuity Pathway Analysis (IPA) evaluates the DEGs and predicts activation or inactivation of pathways. A comparative analysis was done to evaluate the canonical pathways that are activated/inactivated across all subsets in both species using IPA. Pattern recognition receptors (PRRs) are the first line of defense against any pathogen. RIG-I-like receptors (RLRs): RIG-1, LGP2, and MDA-5 that sense viral infection (19) were found to be predominant in CD4^+^, CD8^+^, and CD14^+^ cell subsets at 5 dpv in goats and in CD4^+^ and CD14^+^ cell subsets of sheep (Fig. 4). This RIG-I recognition of viral RNA induces an antiviral state in cells by phosphorylating the IRFs (20) and regulating NF-κB activity through binding to *Nf-κb1* 3′ untranslated region (3′-UTR) mRNA (21). This activation of IRFs by cytosolic pattern recognition receptors was found to be significant in CD4^+^ cells of goats and was triggered, though not significant in CD4^+^ cells of sheep, and CD8^+^ and CD14^+^ cells of both species (Fig. 4). IRF3 was upregulated in CD4^+^, CD14^+^, CD21^+^, and CD335^+^ cells of goats and in CD335^+^ cells of sheep; IRF7 was upregulated in CD4^+^, CD8^+^, CD14^+^, and CD335^+^ cells of sheep and in all cell subsets of goats (Data Set S1). IRF7 was also identified to be the most prominent upstream regulator across subsets in both species. RNA viruses are also recognized by TLR3 (double-stranded RNA [dsRNA]) and/or by TLR7/8 (ssRNA) (22). At 5 dpv, the role of PRRs in the recognition of viruses was found activated in CD14^+^ cells of both sheep and goats and in CD8^+^ cells of goats. TLR2 and TLR4 were upregulated in CD14^+^ cells of both sheep and goats and in CD8^+^ cells of goats. This TLR signaling results in the activation of NF-κB and induction of interferon (IFN)-inducible genes and co-stimulatory molecules (23).

10.1128/mSystems.00820-20.5 DATA SET S1 Individual lists of DEGs expressed in the subsets of goats and sheep. Download Data Set S1, XLS file, 1.2 MB. Copyright © 2021 Wani et al. 2021 Wani et al. https://creativecommons.org/licenses/by/4.0/ This content is distributed under the terms of the Creative Commons Attribution 4.0 International license.

The NF-κB activation by viruses was found activated in CD4^+^, CD8^+^, and CD14^+^ cells of sheep and in CD8^+^ and CD14^+^ cells of goats but was inactivated in CD21^+^ cells of both sheep and goats (Fig. 4). The genes involved in this NF-κB pathway—CD4, LCK, IKK, ERK 1/2, PKR, and RIP—were upregulated in CD4^+^ cells of sheep. LCK, RAS, MEKK1, C-RAF, ERK 1/2, IκB, and CCR5 were upregulated, and CD21 and CXCR5 were downregulated in CD8^+^ cells of sheep. RAS, PKC, ERK 1/2, IκB, NF-κB, and PKR were upregulated in CD14^+^ cells of sheep. CD4, LCK, RAS, PKR, ERK 1/2, and IκB were upregulated and CXCR5 was downregulated in CD8^+^ cells of goats. RIP, PKR, AKT, IKK, ERK 1/2, IκB, and c-RAF were upregulated and CXCR5, CD4, and LCK were downregulated in CD14^+^ cells of goats (Data Set S1). NF-κB acts as a mediator of proinflammatory and anti-inflammatory gene induction and plays a role in regulating T-cell differentiation and effector function (24). Several interleukin and chemokine signaling pathways were found activated in CD4^+^, CD8^+^, and CD14^+^ cells of both species, i.e., interleukin 1 (IL-1) signaling in CD8^+^ cells of goats and CD14^+^ cells of sheep, IL-15 signaling in CD8^+^ cells of sheep and goats, IL-2 signaling in CD8^+^ cells of sheep and goats and CD14^+^ cells of sheep, IL-22 signaling in CD4^+^ cells of sheep and CD8^+^ cells of sheep and goats, IL-6 signaling in CD8^+^ and CD14^+^ cells of sheep and goats, IL-8 signaling in CD4^+^ cells of sheep, and CD8^+^ cells and CD14^+^ cells of sheep and goats, and chemokine signaling in CD8^+^ cells and CD14^+^ cells of sheep and goats (Fig. 4).

**FIG 5:**
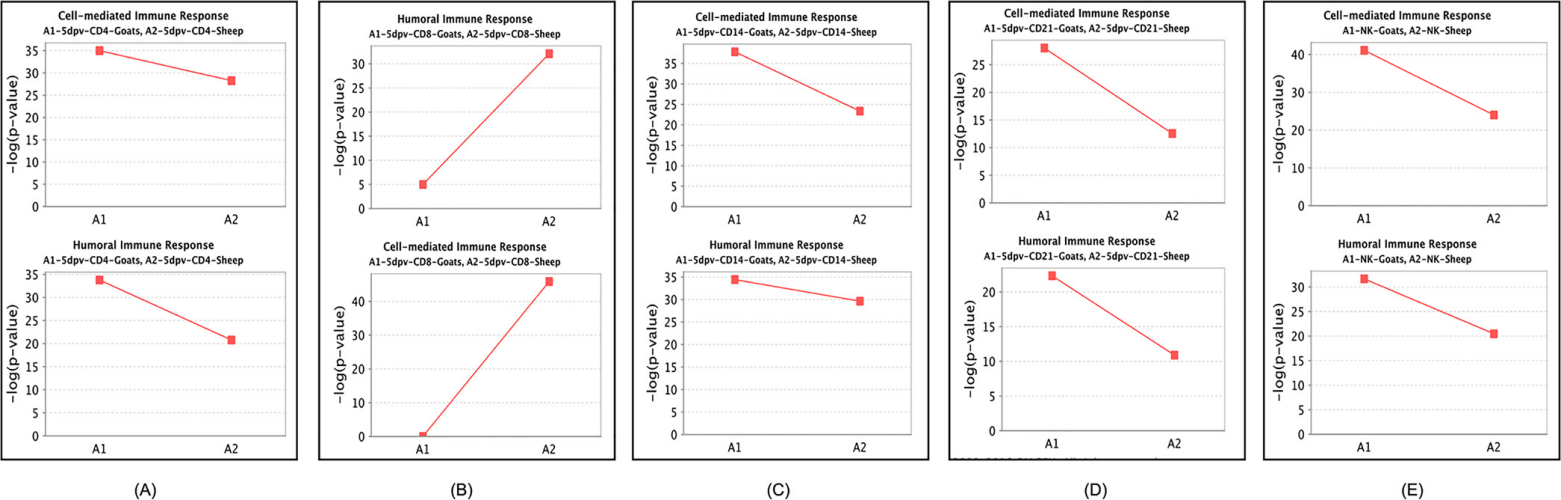
Comparison of significant enrichment (−log *P* value) of genes in cell-mediated immune response and humoral immune response biological functions in CD4^+^ (A), CD8^+^ (B), CD14^+^ (C), CD21^+^ (D) cells and NK cells (CD335^+^) (E) in vaccinated goats and sheep.

Dendritic cell (DC) maturation was found significantly activated in CD21^+^ and CD8^+^ cells of both species. DCs are known to present antigenic peptides complexed with major histocompatibility complex (MHC) class I molecules to CD8-expressing T cells in order to generate cytotoxic cells (25). The interferon signaling pathway that is essential for increased cellular resistance to viral infection was found activated at 5 dpv in CD4^+^, CD8^+^, and CD14^+^ cells of both species. Interestingly, IFN alpha and beta were not dysregulated in any of the subsets in both sheep and goats. The IFN receptors IFNAR1 and IFNAR2 were downregulated in most of the subsets. The absence of expression of type I interferons in our study suggested IFN-independent ISG stimulation as reported previously for PPR (7). However, IFN gamma receptors were found to be activated in most of the subsets. Further, most of the canonical pathways were identified to be inactivated in the CD21^+^ cells. This indicated that the CD21^+^ cells are activated later for the production of antibodies, as a significant increase in antibody production against PPRV vaccination was observed 14 dpv (26). Increase in activity of NK cells (CD335^+^) induces immunoglobulin secretion (27). Here in our study, we observed that the pathways for CD335^+^ cell (NK cell) activity are activated in goats and not in sheep. Also, enrichment (−log *P* value) of genes in cell-mediated immune response and humoral immune response biofunctions was significantly higher in goats than in sheep in CD4^+^, CD14^+^, CD21^+^, and CD335^+^ cells (Fig. 5).

### Protein-protein interaction networks.

**FIG 6:**
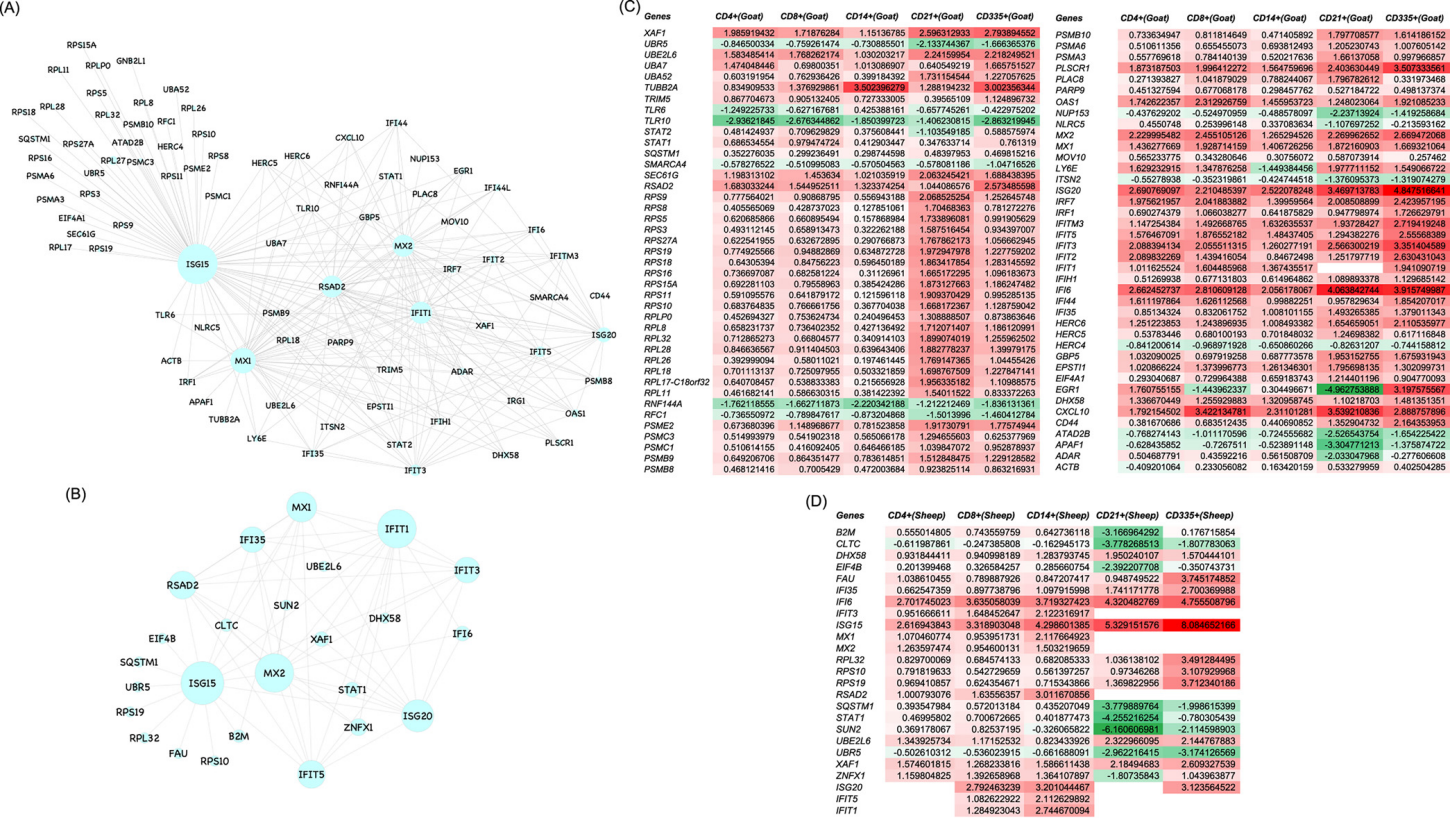
Protein-protein interaction network of antiviral genes IFIT3, ISG15, MX1, MX2, RSAD2, ISG20, IFIT5, and IFIT1 with the common DEGs across all subsets in goats (A) and sheep (B). The size of the circles indicates the degree of interaction. (C) Heatmap for log_2_ fold change (Log_2_FC) values of DEGs involved in the network among the subsets of PBMCs of goats. (D) Heatmap for fold change (log_2_FC) values of DEGs involved in the network among the subsets of PBMCs of sheep. Green color indicates downregulation, and red color indicates upregulation.

The protein-protein interaction network includes hubs connected with interacting genes. The hubs in a network reflect the functional and structural importance of the network. A total number of 618 and 139 DEGs were found to be commonly expressed in goats and sheep, respectively, in all the subsets (Fig. 1). On deciphering the interactions between these DEGs and the eight antiviral molecules (IFIT3, ISG15, MX1, MX2, RSAD2, ISG20, IFIT5, and IFIT1) considered under the knowledge-based approach, most of the antiviral molecules formed the hubs in the network. ISG15 in both species was found to be the major hub with connectivity of 75 and 16 in goats and sheep, respectively (Fig. 6A and B). Heatmap of the genes involved in the networks revealed that most of these antiviral genes in both species are upregulated (Fig. 6C and D).

### Real-time PCR.

The key genes identified from RNA-seq data, *DDX58*, *IFIT3*, *IRF7*, *ISG15*, and *MX1*, were validated by quantitative real-time PCR (qRT-PCR). The expression of all the validated genes was in concordance with RNA sequencing results (Tables 1 and 2).

**TABLE 1:** Log_2_FC from RNA-seq and qRT-PCR of PBMC subsets isolated from Sungri/96 PPRV-vaccinated goats

Gene	CD4		CD8		CD14		CD21		CD335	
	Log_2_FC from qRT-PCR	Log_2_FC from RNA-seq	Log_2_FC from qRT-PCR	Log_2_FC from RNA-seq	Log_2_FC from qRT-PCR	Log_2_FC from RNA-seq	Log_2_FC from qRT-PCR	Log_2_FC from RNA-seq	Log_2_FC from qRT-PCR	Log_2_FC from RNA-seq
DDX58	0.420539	1.163823481	0.093789	1.126889098	1.106442	0.897711159	1.046532	0.866777509
IFIT3	1.315858	2.088394134	0.277734	2.055511315	1.076063	1.260277191	3.739668	2.566300219	3.431138	3.351404589
IRF7	1.677948	1.975621957	1.353633	2.041883882	1.034943	1.39959564	2.719092	2.008508899	2.181422	2.423957195
ISG15	1.954664	3.483488156	2.11479	3.642171919	2.147577	2.749599382	5.468237	4.958535352	4.33885	4.817152957
MX1	0.523217	1.436277669	0.360241	1.928714159	1.459964	1.406726256	2.777281	1.872160903	1.462453	1.669321064

**TABLE 2:** Log_2_FC from RNA-seq and qRT-PCR of PBMC Subsets isolated from Sungri/96 PPRV-vaccinated sheep

Gene	CD4		CD8		CD14		CD21		CD335	
	Log_2_FC from qRT-PCR	Log_2_FC from RNA-seq	Log_2_FC from qRT-PCR	Log_2_FC from RNA-seq	Log_2_FC from qRT-PCR	Log_2_FC from RNA-seq	Log_2_FC from qRT-PCR	Log_2_FC from RNA-seq	Log_2_FC from qRT-PCR	Log_2_FC from RNA-seq
DDX58	1.922081	1.443982294	1.990638	1.880321932	2.506	1.630166862
IFIT3	0.705673	0.951666611	1.950929	1.648452647	2.54733	2.122316917
IRF7	2.137333	2.174678699	2.183255	2.320513832	3.455975	3.017430161	3.74812	3.669000639
ISG15	2.556653	2.616943843	2.495477	3.318903048	3.487653	4.298601385	4.214969	5.329151576	6.072312	8.084652166
MX1	1.83801	1.070460774	1.299464	0.953951731	2.606229	2.117664923

## DISCUSSION

Vaccines protect against an infectious agent by inducing cells or molecules capable of rapidly controlling their replication or by inactivating their toxins. Primarily, vaccines trigger an inflammatory reaction, mediated by cells of the innate immune system: dendritic cells, monocytes, and neutrophils. These cells recognize pathogen-associated molecular patterns (PAMPs) through pattern recognition receptors (PRRs) to become activated to produce cytokines and chemokines (28–31). This inflammatory microenvironment is essential for the differentiation of monocytes to macrophages and the activation and migration of dendritic cells into the draining lymph nodes (32). In the absence of this inflammatory response, the dendritic cells remain immature, and the naive T cells in the lymph nodes do not differentiate into CD4^+^ T cells. PPRV Sungri/96 live attenuated vaccine triggers activation of the innate immune system after it is phagocytosed by monocytes/dendritic cells at the site of administration (33). This RNA virus may be then recognized by TLR3/7 on the endosome or by the RIG-1 or MDA5 in the cytosol to induce an inflammatory response. This induction of inflammatory response is evident in both sheep and goats with the triggering of several pathways *viz.* role of RIG1-like receptors in antiviral innate immunity, the role of pattern recognition receptors in recognition of viruses, production of nitric oxide and reactive oxygen species in macrophages, NF-κB activation by viruses, and several IL signaling pathways in CD14^+^ cells. This triggering in the inflammatory response is much needed for the activation of dendritic cells and monocytes and for further draining of these cells to the nearest lymph node where naive T cells are activated (32). This activation of T cells is clearly seen by the activation of pathways in CD8^+^ (T-cytotoxic) and CD4^+^ (T-helper) cells.

Out of the several pathways activated in both CD4^+^ and CD8^+^ cells, the NOD-like receptor signaling pathway, Th1 and Th2 cell differentiation, T-cell receptor signaling pathway, and Th17 cell differentiation were found to be significantly enriched in both species. The differentiation of T cells to Th1 and Th2 is crucial for inducing the immune response. Th1 cells stimulate cellular immune response, participate in the inhibition of macrophage activation, and stimulate B cells to produce IgM and IgG1 (34). Th2 stimulates humoral immune response, promotes B-cell proliferation, and induces antibody production (34). The distinct subsets of helper T cells, Th1, Th2, and Th17, are effective at protecting against pathogens (35). Additionally, activation of C-type lectin receptor (CLR) signaling in CD8^+^ cells of Sungri/96 vaccinated sheep and goats and in CD4^+^ cells of goats indicate induction of adaptive immune response. C-type lectin receptors are important pattern recognition receptors involved in recognition and induction of adaptive immunity to viruses (36). The significant enrichment of Th1 and Th2 pathways in CD4^+^ cells of goats and sheep in the present study can be correlated with the upregulation of IL-2 (Th1 response) and IL-4 (Th2 response) in our previous study (37). Also, Patel et al. (38) reported that the PPR vaccinated animals showed both Th1 and Th2 response in the initial stage (1 to 5th day) of vaccination. The shift toward Th2 was observed only between 12 and 14 days postvaccination, indicating a bias toward Th2 only at later stages. This corroborates our findings wherein no bias was observed with significant enrichment of Th1 and Th2 pathways in CD4^+^ cells of goats and sheep, and activation of Th1 pathway in CD8^+^ cells of goats and Th2 pathway in CD8^+^ cells of sheep. In CD21^+^ cells, most of the pathways were found inactivated/not activated, as 5 dpv may be too early a time point to detect activation in the CD21^+^ cells and that a significant increase in antibody production against PPRV vaccination was observed at 14 dpv (26).

NK cells (CD335^+^) are known to mediate both innate immune and adaptive immune responses by modulating both CD8^+^ and antibody production (39). In this study, most of the pathways, interferon signaling, cross talk between dendritic cells and natural killer cells, chemokine signaling, inflammasome pathway, inducible nitric oxide synthase (iNOS) signaling, and complement system in NK cells, were found activated in vaccinated goats compared to sheep. Upregulation of RIG-1 and MDA5 in NK cell of goats reflects setting off the innate immune response (40). Also, activation of interferon signaling pathway in infected NK cells of goats suggests evoking of both the innate and adaptive immune responses (41). The activation of iNOS signaling invokes immune response in virus-infected cells (42).The activation of the complement system in NK cells aids in antibody production by bridging both innate and adaptive immune responses (43). Upregulation of CD69, NKp30, FAS, and TNFR2 and activation of cross talk between dendritic cells and natural killer cells in goats must be embarking innate immune response, followed by an adaptive response on antigen presentation after vaccination. The activation and triggering of several pathways in NK cells of goats at this early time point may be because the Sungri/96 vaccine strain is of goat origin and that the activation of these pathways at a later time point in sheep cannot be ruled out.

The network of antiviral molecules (IFIT3, ISG15, MX1, MX2, RSAD2, ISG20, IFIT5, and IFIT1) with the DEGs commonly expressed in the subsets in both sheep and goats, reflected ISG15 as a major hub. The network was found to be dense in goats in comparison to sheep. ISG15 is one of the most highly induced ISGs in viral infections (44, 45), and it has also been found to be directly induced by IRF3/IRF7, independent of IFNs (46–48). It is a ubiquitin-like protein that covalently attaches to target proteins in a process known as ISGylation (44, 49). HERC5 is considered the major ligating enzyme in ISGylation. This ISGylation of viral proteins was reported to have an inhibitory effect on the viral infection (50), whereas ISGylation of host proteins leads to either activation (50) or an increase in the stability (51). HERC6 instead of HERC5 is considered the major ligating enzyme in mice (52). In our study, HERC5 and HERC6 were found upregulated in goats. Further, the antiviral gatekeeper MX1 acts prior to genome replication at an early postentry step of the virus life cycle. Similarly, MX2 specifically targets viral capsid and affects nuclear entry of HIV-1 (44, 53–55). The IFIT family (IFN-induced protein with tetratricopeptide repeats) is a group of ISGs that inhibit virus replication by binding and regulating the functions of cellular and viral proteins and RNAs (56). IFITs were also characterized to play a critical role in protecting hosts from viral pathogenesis. RSAD2, also known as Viperin, is another most highly induced antiviral effector found in endoplasmic reticulum (ER) and ER-derived lipid droplets (57). RSAD2 was characterized to have various modes of antiviral action to inhibit enveloped viruses (58). It can also affect the virus life cycle at an early stage by inhibiting RNA replication (59). All these genes, MX1, MX2, IFIT1, RSAD2, IFIT3, and IFIT5, were found upregulated in both sheep and goats, suggesting a strong antiviral response in both species.

It is important to note that in our study both sheep and goats survived PPRV virulent virus challenge postvaccination, indicating an adequate immune response to counter the virus. In an independent study, it was reported that Sungri/96 vaccine is equally potent in both sheep and goats (60). In our study though, there are a few differences in the systems biology across cells (especially the NK cells) between sheep and goats. The coordinated response that is inclusive of all the cell subsets was found to be toward induction of strong innate immune response, which is needed for an appropriate adaptive immune response.

## MATERIALS AND METHODS

### Animal experiment, ethics statement, and virus.

Live attenuated PPR vaccine virus (Sungri/96) was used as vaccine virus. Permission for studies on animal subjects was obtained and protocols approved from Indian Veterinary Research Institute (IVRI) Institutional Animal Ethics Committee (IAEC) under CPCSEA, India vide letter no. 387/CPCSEA. The vaccine potency testing experiment was carried out per the guidelines of *Indian Pharmacopeia 2014* (61).

In this study, healthy sheep (*n* = 5; age = 12 months) and goats (*n* = 5; age = 12 months) confirmed negative for PPRV antibodies (competitive ELISA [c-ELISA] and serum neutralization test [SNT]) and PPRV antigen (sandwich ELISA) (62, 63) were used. On c-ELISA, the samples with a percent inhibition (PI) value of >40% were considered positive. The animals were acclimatized for 14 days, followed by vaccination on day 0 with a 10^3^ 50% tissue culture infective dose (TCID_50_) field dose of Sungri/96 strain through the subcutaneous route, as mentioned in our previous report (37). All the animals vaccinated survived the challenge from the virulent PPRV in the vaccine potency testing experiment.

### Isolation of T helper cells, T cytotoxic cells, B lymphocytes, monocytes, and natural killer cells by MACS technology.

Blood samples were collected from the animals (*n* = 5) of both species in heparin-coated vacutainer vials at 0 day (just before vaccination) and 5 days postvaccination (5 dpv). PBMCs were isolated by using the Ficoll Histopaque gradient method. PBMCs were strained through cell strainer of 0.40-μm size. The PBMC cell subsets were enriched by positive selection using indirect magnetically activated cell sorting (MACS) technology (Miltenyi Biotech). Cell sorting was done per the manufacturer’s protocol. Initially, the cell-specific surface marker fluorescein isothiocyanate (FITC)-conjugated primary antibodies, anti-CD4^+^ (T helper cells; MCA2213F), anti-CD8^+^ (T cytotoxic cells; MCA2216F), anti-CD14^+^ (monocytes; MCA1568F), and anti-CD21^+^ (B lymphocytes; MCA1195F), were used. For CD335 (NK cell), anti-CD335^+^ (MCA5933GA as the primary antibody) and FITC-labeled secondary antibody (F9137) were used. Subsequently, the cells were magnetically labeled with anti-FITC MicroBeads. Then the cell suspension was loaded on a miniMACS column which was placed in the magnetic field of a MACS Separator. The magnetically labeled cells were retained in the column, while the unlabeled cells run through. After removal of the column from the magnetic field, the magnetically retained cells were eluted as a positively selected cell fraction. The purity of the cells was further checked by a flow cytometer (BD Biosciences). The cells were stored in RNA later for further use at −80^°^C. Cells were kept on ice, and cold buffers were employed to minimize alterations in gene expression during labeling and sorting.

### RNA sequencing of the samples.

Total RNA from each of the PBMC subsets was isolated using the RNeasy minikit (Qiagen GmbH, Germany) per the manufacturer’s protocol. The integrity and quantity of isolated RNA were assessed on a Bioanalyzer (Agilent Technologies, Inc.). The library was prepared using NEBNext Ultra RNA Library Prep kit for Illumina (New England Biolabs Inc.) following the manufacturer’s protocol. Approximately 100 ng of RNA from each sample was used for RNA library preparation. The quality of the libraries was assessed on a Bioanalyzer. Libraries were quantified using a Qubit 2.0 fluorometer (Life Technologies) and by qPCR. The library (1.3 ml, 1.8 pM) was denatured, diluted, and loaded onto a flow cell for sequencing. cDNA library preparation and Illumina sequencing were performed at Bioserve Pvt. (Hyderabad, India). RNA sequencing (RNA-Seq) data were generated in FASTQ format.

### Raw data processing.

Raw sequence data from each sample were subjected to quality control checks using FastQC (Babraham Bioinformatics). Low quality reads with a mean phred score less than or equal to 25 and reads shorter in length than 50 bases were removed using prinseq-lite software (64) before downstream analysis.

### Differential expression and identification of differentially expressed genes.

**FIG 7:**
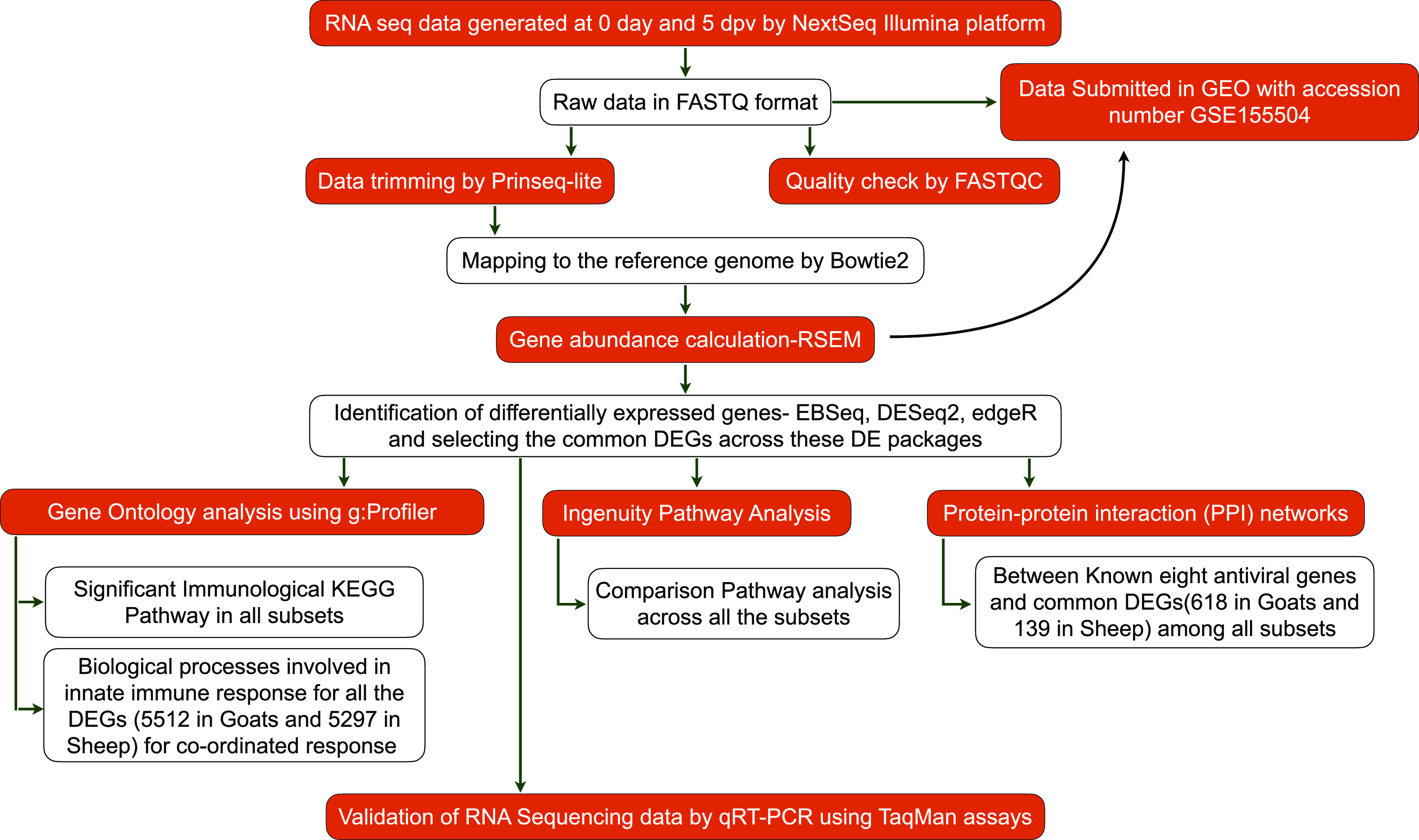
Workflow for RNA sequencing data analysis.

Figure 7 summarizes the steps used in the analysis. Quality filtered reads from control and vaccinated samples (0 day and 5 dpv) were mapped to the Capra hircus or Ovis aries reference genome for the respective subsets. The gene counts were obtained using Bowtie2.0 in RSEM (65). The counts were used for calculating differentially expressed genes (DEGs) by use of R packages—EBSeq, DESeq2, and edgeR. The common DEGs from the three packages were used for downstream analysis while fold changes for the corresponding genes were taken from DESeq2 (66).

### Gene Ontology analysis.

Initially, DEGs of each subset (CD4^+^, CD8^+^, CD14^+^, CD21^+^, and CD335^+^) in sheep and goats were functionally annotated in g:Profiler to identify the significant immune system KEGG pathways. The expression of common DEGs of each subset between sheep and goats that are involved in immunological KEGG pathways is represented in a heatmap. Finally, to understand the coordinated response across all the subsets, genes expressed in all cell subsets were functionally annotated in g:Profiler (a gene is considered expressed if it is expressed in one subset).

### Comparison analysis using Ingenuity Pathway Analysis.

Ingenuity Pathway Analysis (IPA) is an all-in-one, web-based software application that enables analysis, integration, and understanding of data from gene expression, microRNA (miRNA), and single nucleotide polymorphism (SNP) microarrays, as well as metabolomics, proteomics, and RNA-seq experiments. The DEGs from all the subsets in both species were overlaid in IPA against its Ingenuity Knowledge Base (IKB) to perform a comparative analysis. Canonical pathways activated (Z score > 2) or inactivated (Z score < −2) across all the subsets were identified. Also, comparison analysis of subsets in both species for diseases and biofunctions was done to identify the pathways with significant enrichment in the subsets which is based on *P* value.

### Protein-protein interaction networks.

Using a knowledge-based approach (44, 67), antiviral genes IFIT3 (interferon-induced protein with tetratricopeptide repeats 3), ISG15 (interferon-stimulated gene 15), MX1 (MX dynamin-like GTPase 1), MX2 (MX dynamin like GTPase 2), RSAD2 (radical SAM domain-containing 2), ISG20 (interferon-stimulated gene 20), IFIT5 (interferon-induced protein with tetratricopeptide repeats 5), and IFIT1 (interferon-induced protein with tetratricopeptide repeats 1) were selected based on their expression in at least one subset. The protein-protein interactions between these antiviral molecules and the common genes among all the subsets for each species were extracted using STRING (68) and customized scripts. The degree or connectivity between the nodes of the network was calculated using igraph package (69). The complete interaction networks were visualized in Cytoscape 3.8.0 (70).

### Validation of DE genes by quantitative real-time PCR (qRT-PCR).

qRT-PCR was performed using Applied Biosystems 7500 Fast system to validate the expression of key genes using glyceraldehyde-3-phosphate dehydrogenase (GAPDH) and 18S rRNA as endogenous controls by TaqMan chemistry in PBMC subsets. GAPDH and 18S rRNA were employed as the internal controls as these were found to be suitable endogenous controls in earlier studies in PPR (71). Key genes used in the study for validation by qRT-PCR are DDX58 (DExD/H-box helicase 58), IFIT3, IRF7 (interferon regulatory factor 7), MX1, ISG15, GAPDH, and 18S rRNA. All the samples were run in triplicates. The relative expression of each sample was calculated using the 2^−ΔΔCT^ method with control as calibrator (72).

### Data availability.

All raw sequencing data generated in this study are available in GEO, NCBI under accession number GSE155504.

## Supplementary Material

Reviewer comments
