# Supplementary figures and images for: Systems Biology behind Immunoprotection of Both Sheep and Goats after Sungri/96 PPRV Vaccination
Source: mSystems. 2021 Mar 30;6(2):e00820-20. doi: 10.1128/mSystems.00820-20 (PMC8546983; doi:10.1128/mSystems.00820-20)

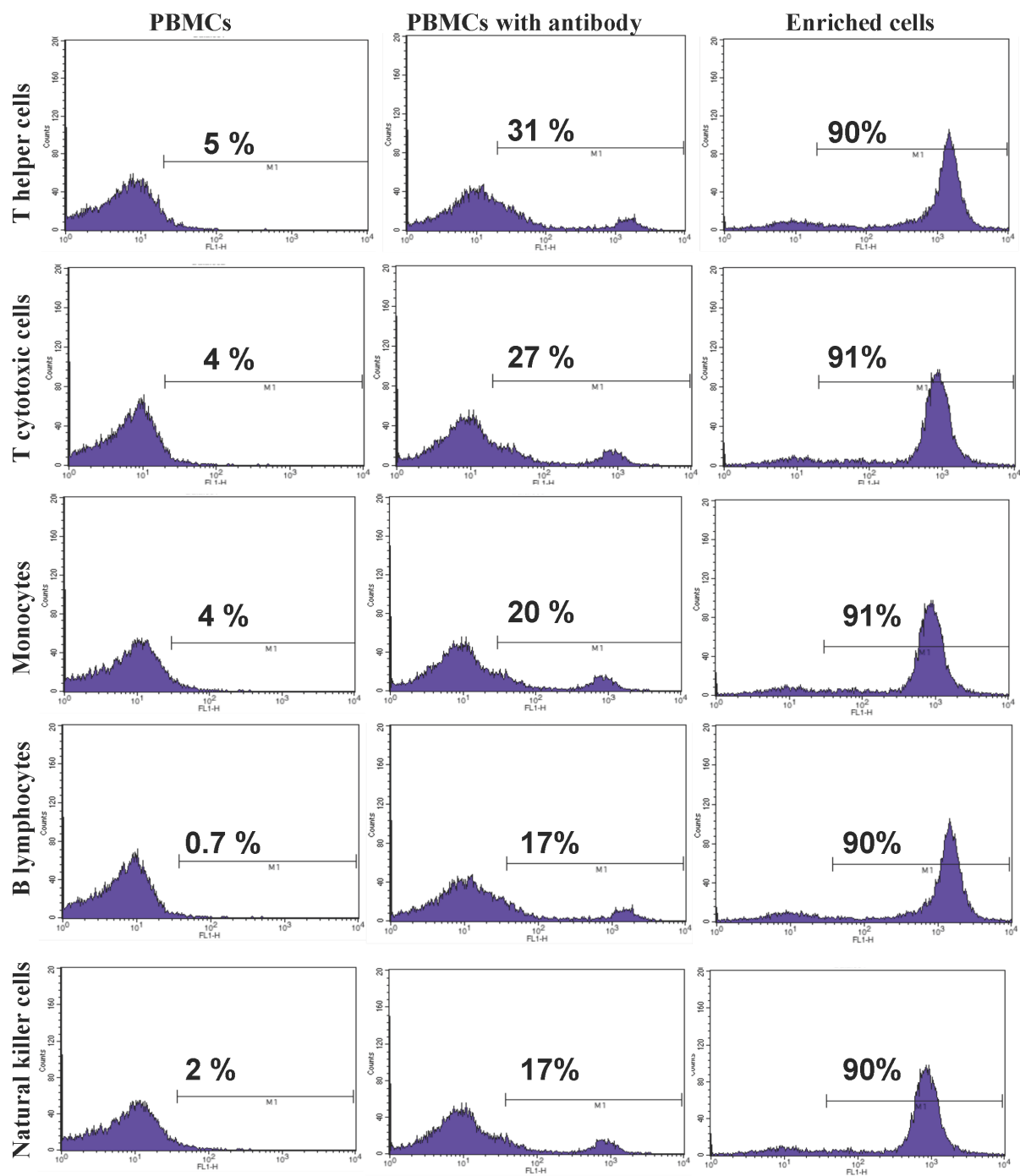

Supplement: FIG S1 [file msystems.00820-20-sf001.pdf]
