# Supplementary material for: Systems Biology behind Immunoprotection of Both Sheep and Goats after Sungri/96 PPRV Vaccination
Source: mSystems. 2021 Mar 30;6(2):e00820-20. doi: 10.1128/mSystems.00820-20 (PMC8546983; doi:10.1128/mSystems.00820-20)
Supplement: FIG S2 [file msystems.00820-20-sf002.pdf]

Pathways	CD4+(Goat)	CD4+(Sheep)	CD8+(Goat)	CD8+(Sheep)	CD14+(Goat)	CD14+(Sheep)	CD21+(Goat)	CD21+(Sheep)	CD335+(Goat)	CD335+(Sheep)
Fc epsilon RI signaling pathway	1.31911082			1.484818163						
Chemokine signaling pathway	2.84451923		2.29127382	4.264383892	2.034020074	1.983479549			3.841598444	1.351409377
Toll-like receptor signaling pathway	3.07682439	2.319475313			2.200457351				3.514357281	1.400736865
TNF signaling pathway	3.16491467				1.748045917	3.671874677	1.692408627		3.072868589	2.801013866
Fc gamma R-mediated phagocytosis	3.51701287	1.592791559	2.9053782	3.469011394	3.208804835	5.294241929	4.23690194		4.530301683	3.0123277
NF-kappa Bsignaling pathway	4.29905412									
NOD-like receptor signaling pathway	4.31895257	4.681281403	3.67477383	2.049533353	3.422540131	6.387703115	2.768087048	1.472601436	6.504155554	1.86244847
C-type lectin receptor signaling pathway	6.11977532		4.39269281	2.939904226	7.614761709	7.3656548			8.321907617	2.249373857
T cell receptor signaling pathway	6.24215039	4.920323412	1.57251622	12.01147857						
Th17 cell differentiation	7.34646832	6.665691379	4.39269281	10.34420962	7.017142306	2.056910869			9.535621268	2.633251439
Th1 and Th2 cell differentiation	8.19656448	7.152128066	3.18482546	9.737308399	6.334973916				7.900689832	
NF-kappa B signaling pathway			1.35676347	1.385860089	4.263988331	1.937196529	1.522038314		6.272076074	2.750182141
Necroptosis			2.23218034						1.331452725	1.416434883
Antigen processing and presentation			3.02898813	3.531324076		1.83289526				
Rap1 signaling pathway				1.504356404	1.315342873					
MAPK signaling pathway				1.708427997					1.383893669	1.48403319
Phagosome					5.767376687					
mTOR signaling pathway							2.836782469			
FoxO signaling pathway							3.633495756		1.742289853	
B cell receptor signaling pathway							7.31692947	1.414921377		
