# Supplementary material for: Systems Biology behind Immunoprotection of Both Sheep and Goats after Sungri/96 PPRV Vaccination
Source: mSystems. 2021 Mar 30;6(2):e00820-20. doi: 10.1128/mSystems.00820-20 (PMC8546983; doi:10.1128/mSystems.00820-20)
Supplement: FIG S3 [file msystems.00820-20-sf003.pdf]

(A) CD4+

Gene	CD4(Goat)	CD4(Sheep)
ACTB	-0.4092011	-0.1613118
ADORA2B	-1.6902583	-1.6070485
AFDN	0.45567737	0.5855647
ARAP3	2.1901951	-1.5598636
CARD6	-0.7350702	-0.6243836
CARD9	1.20508182	0.67241986
CD247	0.86597384	0.76080396
CD3E	0.50124297	0.35011994
CD3G	0.42731213	0.32663895
CD4	0.36198554	0.46123427
CSF1R	1.70722251	-1.875335
CTLA4	1.78751774	1.18493813
CTNNB1	-0.3300979	-0.6529601
CTSB	0.59429203	0.44422741
DLG1	-0.5847158	-0.6278768
ERBIN	0.26862338	0.4014023
EVL	0.33412791	0.29939231
F2R	0.69939735	1.08163581
FOS	4.97331779	3.62422252
FYB1	0.7846191	1.04290009
FYN	0.62771759	0.58176342
GATA3	0.47755056	0.57233945
IKBKB	0.3299542	0.34716616
IL12RB1	0.44992607	0.56419686
IL21R	0.73132282	0.89900593
IL27RA	0.53459879	0.56057007
IL4R	0.89639518	0.51870773
IRF7	1.97562196	2.1746787
ITGAM	-0.5682451	-0.5643886
ITGB2	0.25770271	0.26034674
ITPR1	-1.1526466	-0.8605784
ITPR2	-1.0496846	-0.8072511
JAK3	0.27332273	0.39737408
JUN	3.41327617	4.00315775
LAT	0.626417	0.45604542
LCK	0.60590153	0.54097748
LCP2	0.51285163	0.41627053
MAP3K7	-0.8202005	-0.6635035
MTOR	-0.4218969	-0.3985439
NAIP	1.23296321	-2.733438
NCK2	0.90701028	1.25037311
NFKBIA	1.10995975	0.87972837
NLRP1	1.3821541	-2.8852775
NOTCH2	-1.2593517	-0.9683892
PIK3CA	-0.6562817	-0.4597725
PIK3CD	0.56958912	0.4125489
PIK3R1	-0.6421641	-0.3905242
PKN1	0.53324807	0.59214073
PLCB2	0.53056737	0.48112747
PLCG1	0.70332156	0.41906078
PRKD3	-0.8615356	-0.4423627
PSTPIP1	0.89816102	0.75819755
RASGRP2	0.60381424	0.50881175
RBCK1	0.3850073	0.51842691
RGS14	0.3648515	0.3568524
RIPK1	0.26413441	0.27201866
RUNX3	0.47998382	0.61923827
SIPA1	0.37537911	0.44444204
STAT1	0.68653455	0.46995802
STAT2	0.48142494	0.48467863
STAT5A	0.29909238	0.52619857
TBX21	-1.0854173	-0.6770958
THBS1	2.72150018	-0.9274736
TNFAIP3	1.10701034	2.130349
TXN	0.68487515	0.75393174
TXNIP	0.44002855	0.75518325
ZAP70	0.6666485	0.5229533

(B) CD8+

Gene	CD8(Goat)	CD8(Sheep)
ACTG1	0.44988203	0.17224482
ADCY7	-0.332598	0.30047339
ADORA2B	-1.4917454	-2.2282219
APBB1IP	0.46057246	0.27458923
ARF6	0.36152977	0.39329455
ARHGEF12	-0.8751948	-0.9092307
ARPC1B	0.81833601	0.40629467
ARPC3	0.58412201	0.5985492
ARPC5	0.70743296	0.57211186
ARRB2	0.47446073	1.21326927
ATF4	0.33975751	0.52792474
BCL3	0.82234859	1.03415694
BIRC3	-0.303498	-0.415506
CALM3	0.79634773	0.65589423
CCL5	0.73246346	1.12022433
CD247	0.53277268	0.68460624
CD3D	0.63494745	1.01649553
CD3E	0.42958939	0.98209912
CD3G	0.73845073	0.91523712
CD8A	0.74571062	1.18174615
CSF1	1.61761219	1.81716713
CXCR4	1.49105408	2.63719249
CXCR5	-1.6212257	-1.3817393
F2R	0.8096044	1.82971277
FOS	2.50680073	1.76178776
FYB1	0.58861832	1.26811472
FYN	0.54073471	1.37956904
GABARAPL2	0.34699348	0.47086885
GRK2	0.34957123	0.40132676
GRK3	0.84735606	0.52173914
HLA-DMA	0.5194081	-0.6129386
HLA-DOA	-0.8634142	-1.2165898
HLA-DRA	0.56886089	-0.3063625
HSP90AA1	0.35380925	0.29977806
HSP90AB1	0.22081741	0.26229769
IL27RA	0.263249	0.54481861
IL2RB	0.26920088	0.98262337
IL4R	0.62238505	0.78452508
IRF1	1.06603828	0.56562278
IRF7	2.04188388	2.32051383
IRF9	0.43272056	0.4777884
ITGAL	0.14043814	0.7675066
ITGB1	0.30316629	0.51288732
ITGB2	0.60824526	0.65108082
ITPR1	-0.8235577	-1.2646044
ITPR2	-1.1324604	-0.4799592
ITPR3	-0.9274941	-0.5189484
JAK1	0.22549567	0.56097809
JUN	0.55785974	0.84862982
JUNB	0.25263112	0.80797251
LCK	0.29264622	1.13193024
LYN	0.31305917	-0.4280143
MAP2K1	0.39207819	0.53667826
MAPK3	0.55729687	0.62827712
MAPKAPK2	0.55616064	0.75092569
NCK1	0.58434404	0.91476073
NCK2	1.0026303	1.00283093
NFKBIA	1.82702873	0.81503364
NOTCH2	-0.3882011	-0.8206529
PIK3CA	-0.4643258	-0.7181462
PIK3CD	0.44901466	0.64798374
PKN1	0.17312606	0.50516081
PLCG2	-0.7541967	-0.7607969
PLK3	1.49149527	2.34060347
PREX1	0.26471814	0.58586206
PRKD3	-0.5595984	-0.28837
PSTPIP1	0.36100149	0.99981422

Gene	CD8(Goat)	CD8(Sheep)
PTPRC	0.15655007	0.59176206
RAF1	0.36006871	0.45557501
RAP1B	0.49101241	0.66357272
RASGRP3	-1.0145796	-0.7987131
RBCK1	0.54132337	0.39210672
RHOA	0.61252098	0.45450619
RUNX3	0.56934313	1.16932695
SIPA1	0.32941045	0.75917812
STAT1	0.97947472	0.70067267
STAT2	0.70962983	0.52834857
STAT5B	0.33786343	0.44484438
SYK	-0.5031628	-0.9308284
TGFB1	0.37926473	0.72117573
THBS1	3.10766997	1.27598479
TLN1	0.18544739	0.42988603
TNFAIP3	1.85408906	4.13190823
TNFRSF1B	1.27798214	1.25980698
TRPM2	-0.3579508	0.96376872
TXN	0.941905	0.56303509
TXNIP	0.60841975	1.10153464
VASP	0.70710465	0.46655675
VAV2	-0.9355722	-0.6775842
VDAC2	0.61621629	0.39690712
ZAP70	0.27056493	0.75709597

(C) CD14+

Gene	CD14(Goat)	CD14(Sheep)
ANTXR2	0.5327868	0.33587797
ARF6	0.55238681	0.52050848
ARPC1B	0.65320813	0.62529594
ARPC3	0.35579578	0.69812579
ARPC5	0.73957726	0.53831518
ATF4	0.39353701	0.72339918
ATM	-0.8683729	-1.23101246
BCL2A1	1.22589509	1.47989716
BCL3	0.90048623	1.70590565
BIN1	0.41876682	0.82009876
BIRC3	-0.7810361	0.32105039
CALM2	0.72297769	1.1929394
CALR	0.62487149	0.43440454
CAPN1	0.36889146	0.29721511
CAPN2	0.62667597	0.68817425
CARD6	-0.6355431	-0.62595479
CARD9	0.6386606	0.63306682
CD14	1.55106963	1.50018099
CHMP2A	0.83883051	0.64508827
CHMP4B	0.38689586	0.44336317
CIITA	-0.5215427	-0.64663213
CLEC4D	0.64553848	0.52747448
CSF1	2.07666552	1.09054739
CTSB	0.79114064	0.64474754
CTSS	0.83936503	0.63068464
CXCL10	2.31101281	2.41445002
CXCL8	4.89060415	6.88325684
CYBA	0.82542501	0.83255099
DDX58	0.71771116	1.63016686
DNM2	0.4388749	0.31760675
EGR2	2.63276576	2.78904601
EGR3	3.26548009	3.29950851
EIF2AK2	0.43198358	1.01626687
ERBIN	0.25476386	0.26666357
FCER1G	0.66046173	0.30099296
FOS	2.38981338	2.39601751
FTH1	0.79527917	1.18608987
GAB2	0.88744504	0.63599767
GABARAP	0.33043837	0.45590056
GABARAPL2	0.29607795	0.70192599
GBP5	0.68777358	0.6099444
GLUL	1.40608204	0.50040394
GSDMD	0.71602828	0.58194023
GSN	0.44899844	0.50364262
H2AFJ	0.83767174	1.26689969
H2AFZ	0.55555967	0.80091828
HCK	0.36320594	0.32983218
HIF1A	0.95756073	1.35555084
HLA-DOA	-0.9599719	-1.09286221
HLA-DOB	-1.985783	-0.86888234
HLA-DRA	0.40197986	-0.30126821
HSP90AA1	0.38298494	0.32230615
HSPA5	0.81833997	1.01363006
ICAM1	1.37667272	1.87753144
IFI30	0.86404205	1.14480544
IFNGR1	0.24995045	0.57266073
IFNGR2	0.80156752	0.62742754
IL10	2.89823353	4.0346889
IL1B	3.58017087	4.90458596
IL1RAP	0.66075102	0.80321172
IL21R	-1.3105845	2.80566848
IL4R	0.91932279	1.14750479
IL6R	0.54240451	-0.16077475
INPPL1	0.48119514	0.58797376
IRF1	0.64187583	1.01102366
IRF7	1.39959564	3.01743016
ITPR1	-1.0139961	-0.94481588

Gene	CD14(Goat)	CD14(Sheep)
ITPR2	-0.6809527	-0.6550451
ITPR3	-1.7338389	-0.95669239
JAG1	1.95749912	1.79854301
JUN	-0.696744	-0.52795794
JUNB	0.72495267	1.82654962
LIMK1	0.96937372	0.46001027
LIMK2	1.59821206	1.08087841
LTBR	0.86344634	0.61891972
LYN	0.50642595	0.48902747
MALT1	-0.8814348	-0.64847383
MAP2K3	0.67541649	0.97370839
MAP3K8	1.3668562	2.72733458
MAPK12	-0.9858121	0.65661109
MAPK3	0.5228382	0.52749403
MAPKAPK2	0.71372613	0.66003633
MYD88	0.86901152	1.08663655
NAMPT	1.1064569	1.02199659
NFKBIA	2.99191986	3.33240352
PARP1	-1.838598	-1.17357614
PDIA3	0.58331482	0.53334398
PIK3CD	0.2957383	0.37480059
PLAU	1.75435657	1.48437456
PLCG1	-1.6114381	-1.48119838
PLK3	3.59168336	4.69645798
PRKCD	0.42951607	0.73380509
PSME1	0.67431023	0.68248909
PSME2	0.78152386	0.80888151
PSTPIP1	0.71197321	0.75659768
PTGS2	1.63230476	1.77869747
PTPRC	0.18323639	0.32026816
PYCARD	0.72087735	0.48425674
PYGL	0.38456071	0.49255408
RAF1	0.54876743	0.68091722
RARA	0.34083753	0.2454338
RBCK1	0.54639303	0.37294764
RHOA	0.6579568	0.53427719
RRAS	0.66481137	1.11995863
SMAD3	-1.6637423	-1.5695447
SQSTM1	0.2987446	0.43520705
STAT1	0.41290345	0.40187747
STAT2	0.37560844	0.54159904
TAP1	0.66911457	0.8226459
TAPBP	0.32315479	0.27682756
TBX21	-2.8509593	-2.81823037
TGFB1	0.42974243	0.33565105
TLR4	1.66263266	1.94492156
TNFAIP3	2.80421054	2.50973366
TNFRSF1A	1.03645732	1.4194329
TNFRSF1B	0.48554391	0.36752275
TP53BP1	-1.0444156	-1.00857046
TRADD	0.61817098	0.66628729
TRPV2	0.48476611	0.33033604
TXN	0.92197936	1.08919477
VASP	0.73903823	0.72873496
VDAC2	0.55130039	0.51628141

(D) CD21+

Gene	CD21(Goat)	CD21(Sheep)
AKT2	-1.1448228	-3.42408904
CARD11	-0.353808	-3.96225753
CARD9	1.37097904	4.59849522
CD79A	0.94731977	-3.49995998
INPPL1	-0.9711121	-2.33854461
IRF1	0.94779897	-2.72089597
JUN	0.54174135	-1.99797201
NFKB2	0.37556961	-3.70441254
NFKBIA	2.24620096	3.68707203
PIK3AP1	-2.4533243	-3.34829045
PIK3CD	-1.8931375	-1.93013595
PIK3R1	-2.2757899	-3.42827392
PTPN6	0.18139859	-1.66714231
PYCARD	1.05414954	2.99103483
RHOA	0.39227699	-4.05325401
STAT1	0.34763371	-4.25521625
SYK	-0.8473558	-3.51007823

(E) CD335+

Gene	CD335(Goat)	CD335(Sheep)
IKBKB	0.79343338	-2.954676849
IL27RA	0.80335886	-1.008445503
IL4R	0.80462802	-2.682006144
IL6R	-0.7049476	-3.241618246
INPP5D	0.80861512	-2.411506315
IRAK4	-1.37840194	-2.934951538
IRF3	0.81975393	1.181772138
IRF5	0.82005843	-1.457463822
IRF7	0.82253601	3.669000639
IRF9	0.82288484	1.641780772
ITGAL	-0.71000088	-5.039003536
ITGAM	0.82774631	-5.831927496
ITGB1	-0.71011692	-3.458671436
ITGB2	0.8294062	-1.868154627
ITPR1	0.83305166	-3.253501549
ITPR2	0.83337226	-1.823422314
ITPR3	0.83382453	-2.207528456
JAK1	-0.71069224	-5.102664125
JAK2	0.84113366	-2.281276224
JAK3	0.84121816	-3.099970213
JUN	0.84699524	3.848245946
JUNB	0.84769562	3.253935659
LCP2	0.90518949	-0.946262339
LTB	0.94220522	2.251994336
LYN	0.9490137	-2.921252639
MALT1	-1.31746474	-2.201920181
MAP3K5	0.95981311	-1.170671268
MAP3K7	0.96103598	-2.144206732
MAPK12	-1.30839129	2.137121289
MAPK3	-0.73707175	1.097269915
MAPKAPK2	-0.73733424	-1.305219805
MTOR	1.02161194	-1.795881372
MYC	-1.27866522	-1.258993646
NCF1	1.05106499	0.966463941
NCK1	-0.76436704	-2.106080019
NFATC1	-1.26146823	-2.408395429
NFKB1	1.09527113	-4.48168883
NFKBIA	1.09581218	3.964377362
NLRP1	1.10283094	-2.163522034
NOTCH1	1.11103307	-2.61540906
NOTCH2	1.11111828	-5.197312577
PARP1	1.16824714	-2.142910172
PIK3CA	1.24477643	-3.289412438
PIK3CB	1.24505369	-3.242020953
PIK3CD	1.24542357	-3.549282779
PIK3CG	-1.23040222	-3.531750124
PIK3R1	1.24669532	-2.62109678
PIK3R5	1.24764894	-1.339986596
PKN1	1.2559625	1.998400441
PLAU	-0.80423922	1.793549116
PLCG1	1.26575743	-3.951624614
PLCG2	1.26621452	-3.581331669
PREX1	1.33217487	-2.991917586
PRKCB	1.33742227	-3.206635814
PRKCQ	-1.2059523	-2.414428609
PRKD2	-1.20570857	1.759738154
PRKD3	1.3385061	-3.378525999
PSTPIP1	1.39077066	1.199585026
PTGS2	1.40340333	-4.18070619
PTPRC	-0.8358976	-3.549452619
PXN	-1.19341583	-2.559576628
PYCARD	-0.83593514	1.218134659
RAF1	-0.84383977	-1.131793905
RAP1A	-0.84544691	-1.682712373
RAP1B	-0.84581916	-1.415804408
RARA	-1.17678614	-1.170925269
RASGRP2	1.48135135	1.797293537

Gene	CD335(Goat)	CD335(Sheep)
ACTB	-0.47038563	-0.99291063
ACTG1	-0.49023222	-1.80524809
ADCY3	-1.73698526	-1.78962149
ADCY7	-0.49308316	-4.285148693
ADORA2B	-0.44863036	-1.83472718
AKT2	-0.42650426	-1.513935102
ANTXR2	-1.70734569	-1.256746129
APBB1IP	-0.514689	-2.205021388
ARF6	-0.51770765	-1.257970479
ARHGEF12	-0.35436236	-1.317137857
ARPC1B	-0.34258829	1.137893438
ARPC3	-0.52161773	0.774593541
ARPC5	-0.52527729	-3.043699801
ARPC5L	-1.68216509	1.026569214
ARRB1	-0.34185547	-4.360552455
ATF4	-0.31291602	2.116486443
ATF6B	-1.67753483	0.674317455
ATM	-0.29962293	-1.914766001
BIRC3	0.26065384	-0.881527177
BLNK	0.268072	-1.352651851
CALM3	0.33810042	1.203245668
CARD11	-1.64685006	-0.349936052
CARD6	0.35944861	-3.858761527
CARD9	0.36262386	2.818120911
CBL	0.36382542	-2.750709066
CBLB	0.36809924	-3.070426954
CCR4	0.38948447	2.562498273
CD3E	0.40634131	-3.703183369
CD4	0.41055345	-4.410548004
CD86	-1.61693479	-1.624214435
CDKN1B	0.44148619	0.5955617
CSF1R	0.49887377	-3.788867573
CTNNB1	0.51004314	-2.621853435
CTNND1	-0.60219503	2.248043258
CTSB	0.51152228	-4.842592629
CTSK	0.51215377	-2.476655178
CXCR4	0.5172416	1.408148738
CXCR5	-0.60764201	-1.580017947
CYBA	0.52525769	2.568244667
DNM1L	0.57260069	-2.085356415
DNM2	-1.53034574	-2.098386527
EGR2	-1.51526422	-2.992469515
ELMO1	0.6238481	-2.95223418
ERBIN	0.63820457	-2.139107059
F2R	0.65755504	-4.611244332
FARP2	-1.47870057	3.42191228
FGFR2	0.68088024	4.005091747
FGR	0.68772374	-1.387759928
FOS	0.69929449	3.152806726
GAB2	-0.66025311	-1.752292138
GABARAP	-0.66187037	1.397878639
GATA3	0.71668094	1.815977034
GBP5	0.72150998	-1.678049989
GNAI2	-0.67155071	-2.842673728
GNB2	0.72710771	0.77936766
GRB2	-1.42574271	-0.783541252
GRK2	0.73539645	-1.095951513
GRK3	-0.68146442	-2.336975802
GSDMD	0.73663318	1.160894829
GSN	0.73707381	0.751802712
HBEGF	0.74287111	2.63259182
HCK	0.74375456	-0.534327881
HIF1A	-1.41579388	-3.625474796
HLA-DOA	0.7594226	-0.669463369
HLA-DRA	0.75965444	-0.906111983
HSP90AA1	-0.69332405	0.77358541
IFNAR1	0.79231247	-4.989252757

Gene	CD335(Goat)	CD335(Sheep)
RASGRP3	1.48139572	-4.084430773
RELA	1.5146613	-1.647484202
RGS14	1.55146055	-1.37248987
RHOA	-0.85521125	-1.235892032
RIPK1	1.59073734	-0.589460444
ROCK1	1.64536145	-4.257793686
ROCK2	-1.14336633	-3.204963911
RPS6KB2	1.68941618	1.242973592
RRAS	-1.13865021	2.147645081
RRAS2	1.70408976	-2.117234229
SHARPIN	1.87947626	1.76511954
SIPA1	1.89897168	1.102947437
SIPA1L3	1.90242288	-2.196581125
SMAD3	2.03464912	-2.226005844
STAT1	2.24829699	-0.780305439
STAT2	2.25385573	-2.722541723
STAT5A	2.25727347	-1.794015924
STAT5B	2.25728711	-3.614962461
SYK	2.34993195	-1.28674913
TAB1	2.35945584	-3.50200279
TBX21	2.40712682	-1.477114155
TGFBR2	2.49058952	-2.21651577
THBS1	2.51612179	-3.275405662
TLN1	2.56850504	-2.667574993
TLR2	-0.90311005	-1.555465902
TLR4	2.58110767	-1.669800282
TLR6	2.58487009	-1.875377467
TNFRSF13C	-1.03992632	1.58406213
TNFRSF1B	2.66446129	-2.671200696
TP53BP1	-1.03206392	-0.723583749
TRAF1	-3.03234198	-1.203991888
TRAF3	-2.9954657	-1.047541712
TRAF5	-1.02661539	-1.541393223
TRIM25	-0.92198449	-2.243775468
TRPM2	-0.92323301	-3.141875565
TRPM7	-0.92359172	-2.277356222
TRPV2	-1.01969307	2.234964245
TXN	2.93264592	2.555436345
TXNIP	2.9363427	-0.467459041
TYK2	-2.37402689	-2.858854255
VASP	3.29040683	-1.485729853
VAV2	3.30805271	-3.717656816
VAV3	-2.10095573	-2.398662406
VDAC1	-0.99055496	-3.753829037
ZAP70	3.6978087	-1.523088663
